# Microscopical Observations in Yellow Fever

**Published:** 1878-12-20

**Authors:** J. B. Marvin

**Affiliations:** Professor of Chemistry and Microscopy, Hospital Medical College; President of Microscopical Society, Louisville Ky.


### MICROSCOPICAL OBSERVATIONS IN YELLOW FEVER.

BY J. B. MARVIN, M. D.,
Professor of Chemistry and Microscopy, Hospital Medical College; President of Microscopical Society, Louisville, Ky.

The literature of yellow fever is very voluminous, but our knowlege of the pathological anatomy of the disease is very meager. I believe by calling to our aid chemistry and the microscope most valuable additions will be made to our knowledge of the disease. While resident physician at the Louisville, Yellow Fever Hospital I improved my opportunity by making frequent and extended chemical and microscopical examinations. I present you to-night a brief summary of my work as far as I have finished. I present you bare facts, illustrating my remarks with mounted specimens. I offer no theories or deductions from my work, believing that our knowledge of this disease will be best advanced by a careful and conscientious record of facts instead of vagaries and theoretical hypotheses.

*The Breath.*—Pure glycerine was smeared in the center of a clean new glass slide, and held an inch or two from the nostrils or mouth of the patient. After a few minutes' exposure to the breath the slide was examined under the microscope. Large quantities of very active vibrios were revealed; they were of the short, dotted variety. There were also found roundish, oval, moving bodies, probably bacteria.

*The Blood.*—A drop of blood from the finger was received on a slide covered with thin glass, avoiding pressure, and examined. The corpuscles were more or less jagged and crenated. In some severe cases there was a large increase in the number of white corpuscles. Scattered among the corpuscles were small oval and rod-shaped bodies, yellowish in color, and quite active in their movements. They were probably bactria, but do not resemble vibrios or any form of bacteria that I am familiar with. More extended observations in this and other fevers must be made before attaching undue importance to the existence of these bodies in the blood and breath. The question naturally suggests itself whether these bodies are the cause or the result of this disease. I incline to the belief that they are the result. Every precaution was taken, in making these examinations of the breath and blood, to avoid contamination. The examinations were made with a Folles one-tenth inch immersion objective and a "B" ocular.

*The Urine.*—The points of interest in the urine were the constant presence of granular tube casts, renal epithelium, and granular matter, all more or less stained yellow with bile; the tube-casts in severe cases appearing as soon as the second day, more generally on the third or fourth day of the attack. In all cases there was an admixture of vesical epithelium. In some few cases there was a great abundance of vesical epithelium for a few days before the appearance of tube-casts or renal derivatives. The quantity of tube-casts may be small or very large. The severer the case, the greater the quantity of casts. Tube-casts are very valuable guides in the prognosis of the disease. As convalescence sets in, the casts generally disappear. In some cases, however, they continue in considerable quantity till after the patient is up and walking about.

*The Vomit.*—After the stomach had been emptied of food the vomit was glairy mucus and epithelium streaked with blood, bearing a striking resemblance to the sputum of pneumonia. Bile in greater or less quantity was generally present. Frequently pure blood was vomited in large quantities, the ejection of blood frequently alternating and following black vomit. The coffee-ground or black vomit consists of blood more or less digested and broken down by the gastric juice and bile. There were large quantities of vibrios, an oval, not recognized growth, and frequently very large crystals of haematoidin.

*The Liver.*—The principal pathological charges are found in the liver. The color may be bright yellow, orange nutmeg, or normal. The organ is generally enlarged, the enlargement being very slight in some cases. It is very firm and tough. On section the hepatic cells are granular, frequently stained with bile, and have undergone almost complete fatty degeneration. There is generally an increase of the connective tissue and a consequent pressure upon and destruction of the cells. In one case, aged twenty-seven, not a drinker, who had suffered at intervals for two years with malarial fever, there was an enormous increase of the connective tissue visible to the eye, giving to the organ the appearance found in cirrhosis. On section all the appearance of cirrhosis were found, with marked fatty degeneration in parts, and in other places amyloid degeneration.

*The Kidneys.*—The kidneys are congested and in some cases considerably enlarged. On section there are found tubal and inter-tubal hemorrhages. The tubes are filled with granular matter and epithelium; in some parts the tubes are empty and completely denuded of epithelium. There is frequently fatty degeneration, slight in degree. In short, the kidneys present all the appearance ot Bright's disease.

*The Spleen.*—The spleen presents no constant or marked deviation from health. In some cases, which gave history of previous malarial trouble, the organ was enlarged and pigmented. In other cases there was no enlargement or pigmentation.

*The Stomach.*—This organ does not appear congested as stated in text-books. The mucous membrane is pale, and is not destroyed. In only one case was there any thickening of the membrane or enlargement of the rugæ. On section, the glands and villi are but slightly changed. The villi, especially their free extremities, contain blood. I am convinced that the changes stated to have been found in this organ are really post-mortem changes, due to the fact that examinations were not made until some hours after death. Post-mortem changes are very rapid, and the sooner an examination is made the better.

*The Intestines.*—The intestines generally present the same appearance as the stomach. In some cases there is marked congestion, and the villi present appearance of acute catarrh.

*The Bladder.*—In those cases where there is suppression of the urine for any length of time before death, the bladder is badly congested, the mucous membrane being purple in spots. In other cases there is no marked change. The gall bladder is full of bile, frequently greatly distended and badly congested.

*The Lungs.*—This organ presents no constant change. In several cases there was recent pleuritic adhesion; in one case there was severe pneumonia. In some cases the organ is completely collapsed. The color is generally dark and mottled; hemorrhagic spots are frequent.

*The Heart.*—The heart may be full or empty. In some cases there is marked fatty degeneration, the walls being pale and friable. Most generally the organ is normal. The pericardium always contains more or less reddish fluid, the amount varying from one to six ounces.

*The Brain.*—No lesions were found in the cerebrum, nor constant change at the base of the organ. In cases which had marked delirium, there was marked congestion and softening at the base. I have not finished my microscopic examination of this organ.—*Louisville Medical News.*

